# DNA methylation biomarker for cumulative lead exposure is associated with Parkinson’s disease

**DOI:** 10.1186/s13148-021-01051-3

**Published:** 2021-03-22

**Authors:** Kimberly C. Paul, Steve Horvath, Irish Del Rosario, Jeff M. Bronstein, Beate Ritz

**Affiliations:** 1 Department of Neurology, David Geffen School of Medicine, 73-320B CHS Campus, Los Angeles, CA 177220 USA; 2 Department of Human Genetics, David Geffen School of Medicine, Los Angeles, CA USA; 3 Department of Epidemiology, UCLA Fielding School of Public Health, Los Angeles, CA USA

**Keywords:** Parkinson’s Disease, Lead, DNA methylation

## Abstract

Lead, a known neurotoxicant, has previously received attention in Parkinson’s disease (PD) research, but epidemiologic studies have been limited in sample size and findings are equivocal. We generated two methylation-based biomarkers for cumulative tibia and patella bone-measured lead exposure in 1528 PD patients and 1169 controls. PD status was associated with increased levels of the DNAm biomarker for tibia-lead levels. We estimated a meta-OR for PD of 1.89 per unit DNAm tibia-lead increase (95% CI 1.59, 2.24; p = 8.1E−13). The current study supports the notion that chronic and long-term lead exposure tracked via DNAm may contribute to PD pathogenesis.

## Introduction

Parkinson’s disease (PD) is a progressive neurodegenerative disorder characterized by gradual loss of dopaminergic neurons in the substantia nigra. PD etiology is complex and multi-factorial, likely involving a combination of genetic, aging, and environmental components. Lead, a known neurotoxicant, has previously received attention in PD, but findings in epidemiologic studies have been equivocal.

Lead passes through the blood–brain barrier and enters neurons and glia through calcium channels, affecting neurotransmitter release, energy metabolism, and inducing reactive oxygen species (1). Long-term, cumulative lead levels, which depend on both exposure levels and an individual’s uptake, storage, and clearance, may affect the aging brain the most. Interestingly, in epidemiologic studies, results for PD association seem to depend on the type of exposure assessment employed. Specifically, studies using bone-lead measurements found positive associations (2, 3), while those relying on self-report of occupational exposures or emissions data, with one exception (4), did not (5–8). Suggesting that errors in exposure measurement and issues of timing may obscure the role lead plays in neurodegeneration.

Clearly, bone-lead-measurement is the gold-standard for long-term lead exposure, given that the half-life of lead in the tibia is approximately 20 years (9). However, cost and feasibility have often precluded this option, especially in larger studies. Recent advances in high-throughput epigenetic technology are paving the way for biomarker discovery in many fields, including exposure science. Two methylation-based biomarkers for cumulative lead exposure measured in the tibia and patella have recently been developed (10). Using these biomarkers, for the first time we evaluate the influence of methylation-based cumulative lead exposure on PD risk in two, independent population-based studies with over 2600 participants.

## Methods

For our analysis, we used genome-wide DNA methylation (DNAm) data from two publicly available PD studies (Gene Expression Omnibus (GEO) accession numbers GSE145361 (SGPD), GSE72774 and GSE72776 (PEG)). DNAm was measured with the Human Methylation 450 k BeadChip in whole blood samples.

The System Genomics of Parkinson’s Disease (SGPD) is a consortium of three studies from across Australia and New Zealand (11). The data available on GEO data consists of 1889 samples (959 PD patients and 930 controls) of European ancestry. Prevalent PD patients were recruited (PD duration 2–40 + years), and controls consisted primarily of community-based age-matched volunteers from the same communities, as well as some patient’s spouses and siblings. Cohort details, DNA extraction methods, quality control procedures, and normalization methods (quantile-normalized and normalization adjusted for batch, slide, cohort, sentrix row/column, sex, and age) have been described (11).

The Parkinson’s Environment and Genes (PEG) study is population-based study from three agricultural counties of Central California (12). GEO data is available for 807 samples (569 PD patients and 238 controls) of European and Hispanic ancestry. Patients early in disease (mean PD duration = 2.9 years (SD = 2.3)) were diagnosed in-person by UCLA Movement Disorder Specialists (J.B.). Population-based controls from the same communities were randomly sampled from Medicare lists and via residential tax assessor's records. Cohort details, DNA extraction methods, quality control procedures, and normalization methods have been previously described (13).

We generated two epigenetic biomarkers for cumulative lead exposure (tibia and patella), developed in the Normative Aging Study (NAS). The epigenetic biosensors of patella and tibia are linear combinations of 59 and 138 CpGs, respectively, identified with site-by-site analysis and combined via machine-learning algorithms trained on K x-ray fluorescence (KXRF) in-vivo measures of bone-lead (10). To determine lead biomarker levels in SGPD and PEG, we extracted the published regression coefficients from NAS (10), and applied them to the corresponding DNAm beta matrices. At time of publication, the specificity of the DNAm biosensors has not been validated beyond the initial study.

In order to quantify the relationship between the DNAm lead and PD, we used logistic regression to estimate odds ratios (ORs) and 95% CIs for PD. The OR is a measure of association that compares the odds of PD based on exposure, with an OR greater than 1 indicating that the odds of PD increase with exposure. Regression diagnostics showed that model assumptions were met. Based on data availability from GEO, we controlled for age (estimated with the Horvath DNAmAge in SGPD (14)), sex, ancestry (PEG only), blood cell composition, smoking history (PEG only), and mean methylation by sample to account for global methylation. We imputed blood cell proportions using the Houseman method (15). We assessed between-study heterogeneity for SGPD and PEG results with Cochran’s *Q*, and calculated a meta OR using a fixed-effects model with weights based on precision.

## Results

Characteristics of SGPD and PEG participants can be found in Table 1. In both studies, PD patients were a few years older than their respective controls and more often men. In PEG, there were less former/current smokers among patients. Additional file 1: Fig. 1 shows box plots of the data, including all data points and the mean values for both DNAm biomarkers by PD status and stratified by study.

**Table 1:** Study descriptives

Demographic variable	SGPD		PEG	
	PD (n = 959)	Control (n = 930)	PD (n = 569)	Control (n = 238)
Continuous variables: mean (SD)				
Age*	66.5 (9.1)	64.9 (10.4)	70.5 (9.8)	67.5 (12.8)
European fractional ancestry	NA		0.87 (0.27)	0.92 (0.18)
meanMethBySample	0.26 (0.01)	0.27 (0.01)	0.29 (0.01)	0.29 (0.01)
DNAm tibia-lead	2.57 (0.41)	2.46 (0.49)	2.65 (0.54)	2.52 (0.52)
DNAm patella-lead	3.41 (0.4)	3.48 (0.4)	3.06 (0.4)	3.03 (0.3)
Categorical variables: n (%)				
Male	611 (63.7)	428 (46.0)	356 (62.6)	127 (53.4)
Former smoker	NA		240 (42.4)	125 (52.5)
Current smoker			25 (4.2)	17 (7.1)

*For SGPD, age was not provided in the GEO data. To account for age, we therefore generated the epigenetic age based on the Horvath epigenetic clock (DNAmAge, highly correlated with chronologic age R > 0.9 in other studies of older adults)

NA: All participants with SGPD methylation data are of European ancestry (11). Smoking status data not available for SGPD via data not provided in GEO

PD status was strongly associated with the DNAm biomarker for tibia-lead levels (Table 2) in both cohorts (Cochran’s *Q* indicated no significant heterogeneity study results, p = 0.25). Specifically, with minimal adjustment (age, sex, ancestry), we estimated in SGPD, an OR for PD of 2.06 per unit DNAm tibia-lead increase (95% CI 1.66, 2.56; p = 5.4E−11) and in PEG of 1.60 (95% CI 1.20, 2.15; p = 1.0E−03), with a meta-OR of 1.89 (95% CI 1.59, 2.24; p = 8.1E−13). Further adjustment for blood cell composition, mean methylation by sample, and smoking status (PEG only), resulted in a meta OR of 1.52 (95% CI 1.25, 1.86; p = 2.8E−5). The DNAm biomarker for patella-lead levels was inversely associated with PD in SGPD (fully adjusted model: OR = 0.70 (95% CI 0.52, 0.93)), but not in PEG (OR = 1.18 (95% CI 0.80, 1.75) (heterogeneity between study results p = 0.006). Results for both DNAm markers stratified by sex can be found in Additional file 3: Table 1. Additionally, results were similar in models both with or without adjustment for smoking (Additional file 3: Table 2).

**Table 2:** DNAm estimated cumulative lead exposure and PD risk

		SGPD (n = 1889)		PEG (n = 807)		Meta-analysis		Test of study heterogeneity	
		OR (95% CI)	p-value	OR (95% CI)	p-value	OR (95% CI)	p-value	Q	p-value
MODEL 1	DNAm Tibia-lead	2.06 (1.66, 2.56)	5.4E−11	1.60 (1.20, 2.15)	0.001	1.89 (1.59, 2.24)	8.09E−13	1.32	0.25
MODEL 2		1.54 (1.22, 1.95)	2.8E−04	1.48 (1.03, 2.13)	0.019	1.52 (1.25, 1.86)	2.84E−05	1.87	0.17
MODEL 1	DNAm Patella-lead	0.59 (0.45, 0.78)	1.6E−04	1.19 (0.82, 1.73)	0.356	*No replication (study heterogeneity)*		8.83	0.003
MODEL 2		0.70 (0.53, 0.93)	0.015	1.18 (0.80, 1.75)	0.417			7.53	0.006

Model 1: adjusts for age (DNAmAge in SGPD), sex, ancestry (PEG only)

Model 2: adjusts for age (DNAmAge in SGPD), sex, ancestry (PEG only), smoker (PEG only), blood cell composition, and meanMethBySample

## Discussion

In two large population-based PD studies, we used DNAm to estimate cumulative, bone-lead levels by applying an externally developed epigenetic (DNAm) biosensor (10), and found that PD was strongly associated with the DNAm pattern estimated for tibia-lead levels. Positive associations were seen independently in both studies and persisted after multiple adjustments. Our findings for DNAm tibia-lead are supported by positive associations in two prior epidemiologic studies that relied on actual tibia bone-lead measurements but had more limited sample sizes (121 and 330 PD patients (2, 3)).

We found no association between DNAm patella-lead and PD in PEG, and even an inverse-association in the SGPD study. Interestingly, one of the previous epidemiologic studies also only observed tibia- but not patella-lead measurements to be associated with PD (2). This is perhaps due to the half-life of lead, which is approximately 8 years in the patella (representing trabecular bone) versus decades in the tibia (representing cortical bone) (2, 16). Thus, tibia-lead measurements represent cumulative exposure from a more distant past than patella. This shorter half-life may also explain why an inverse-association was observed in SGPD, where the patients had a longer PD duration at blood-draw (2–42 years), while PEG patients were enrolled early in disease (mean 2.9 years). Should a primary source of lead exposure among adults be occupationally related, exposure would be less likely to have occurred among patients in the years around PD diagnosis, as patients stop working several years earlier on average than controls due to disease (17). Industries with occupational lead exposure include construction jobs with lead-based paint activities, including removal or demolition, metal and chemical product manufacturing, battery manufacturing and recycling, and electronics manufacturing (18). Other sources of chronic lead exposure, such as drinking water, leaded gasoline, and household paint, however, may be less influenced by PD onset. Overall, multiple sources of evidence from previous and our current study support a positive association for long-term, cumulative lead exposure that extended over many decades, as measured in the tibia, with PD, rather than shorter-term and more recent exposures reflected in patella-lead levels.

The inordinate advantage of our study is the availability of DNAm-lead biomarkers, which also allows for future applications to all PD studies, or more generally studies of age-related disease, with methylation data and larger-scale systematic research into the long-term neurotoxicity of lead. The technology and methodology to track many health-related factors through methylation is rapidly developing. However, currently the DNAm-lead biomarkers have two limitations in our opinion, first, that they were developed in an all-male cohort, and second, that the specificity has not yet been validated beyond the initial study (10). Future validation of the DNAm-lead biomarkers would be incredibly beneficial to the research community, given the widespread adverse health effects from chronic lead exposure. Our study provides a compelling first glance into the utility of DNAm-lead exposure measures in PD and neurodegenerative research in general, and solid evidence for the involvement of this DNAm-lead exposure pattern in PD pathogenesis.

## Supplementary information

**Additional file 1:** Box plot figures of the DNAm lead-biomarkers by PD and stratified by study.

**Additional file 2:** Table showing DNAm estimated cumulative lead exposure and PD risk, stratified by sex.

**Additional file 3:** Table showing DNAm estimated cumulative lead exposure and PD risk in PEG, with and without controlling for smoking.

## Data Availability

The datasets analyzed during the current study are available in the GEO repository, Accession Numbers GSE145361 (SGPD), GSE72774 and GSE72776 (PEG).
