# Supplementary material for: DNA methylation biomarker for cumulative lead exposure is associated with Parkinson’s disease
Source: Clin Epigenetics. 2021 Mar 22;13:59. doi: 10.1186/s13148-021-01051-3 (PMC7983295; doi:10.1186/s13148-021-01051-3)
Supplement: Supplementary file 1 — Additional file 1: Box plot figures of the DNAm lead-biomarkers by PD and stratified by study. [file 13148_2021_1051_MOESM1_ESM.pdf]

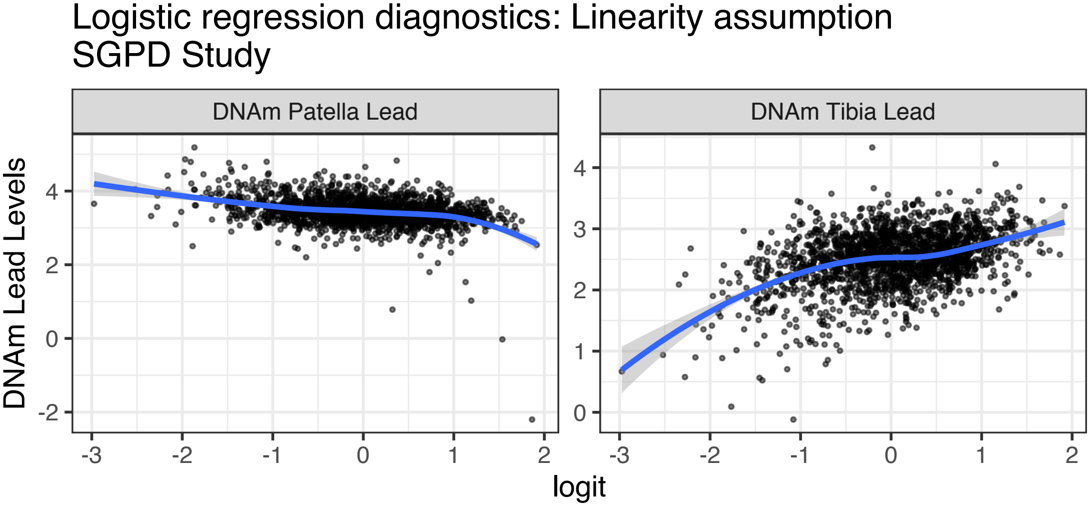
Logistic regression diagnostics: Linearity assumption
SGPD Study
DNAm Patella Lead
DNAm Tibia Lead
DNAm Lead Levels
logit
-3
-2
-1
0
1
2
4
3
2
1
0
-2

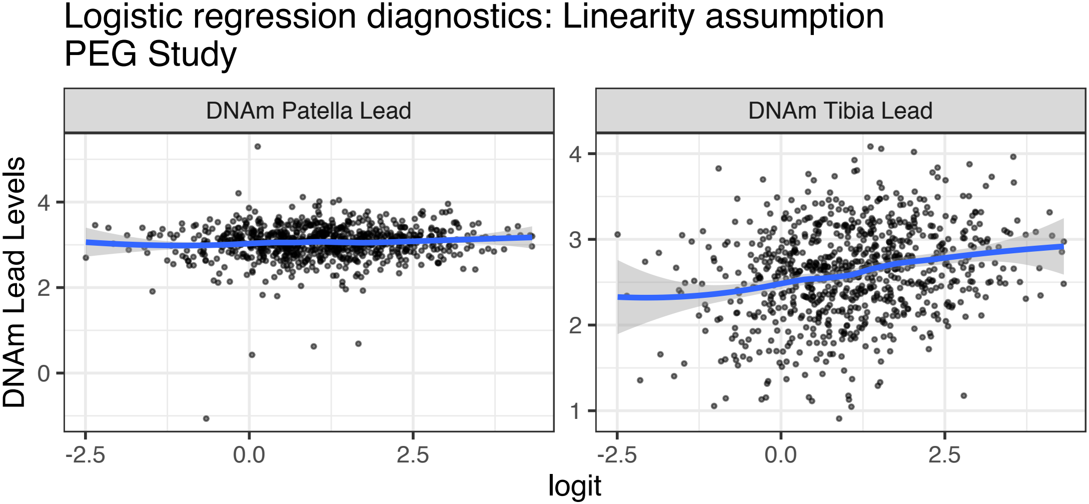
Logistic regression diagnostics: Linearity assumption
PEG Study
DNAm Patella Lead
DNAm Tibia Lead
DNAm Lead Levels
logit
-2.5
0.0
2.5
4
3
2
1
0
