# Supplementary material for: DNA methylation biomarker for cumulative lead exposure is associated with Parkinson’s disease
Source: Clin Epigenetics. 2021 Mar 22;13:59. doi: 10.1186/s13148-021-01051-3 (PMC7983295; doi:10.1186/s13148-021-01051-3)
Supplement: Supplementary file 2 — Additional file 2: Table showing DNAm estimated cumulative lead exposure and PD risk, stratified by sex. [file 13148_2021_1051_MOESM2_ESM.pdf]

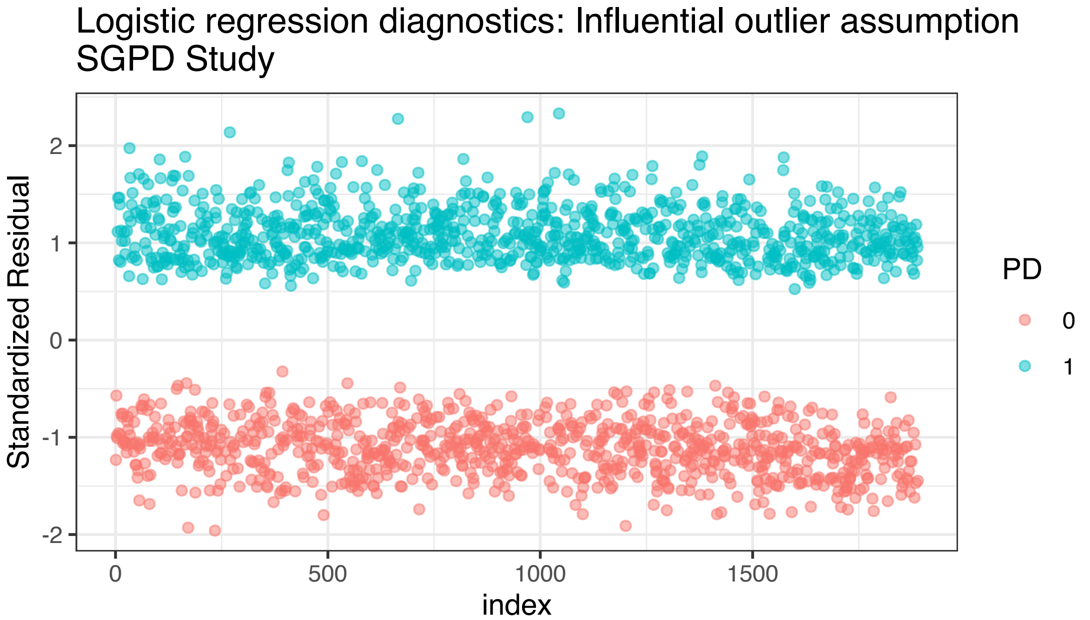
Logistic regression diagnostics: Influential outlier assumption
SGPD Study
Standardized Residual
index
PD
0
1
2
1
0
-1
-2
0
500
1000
1500

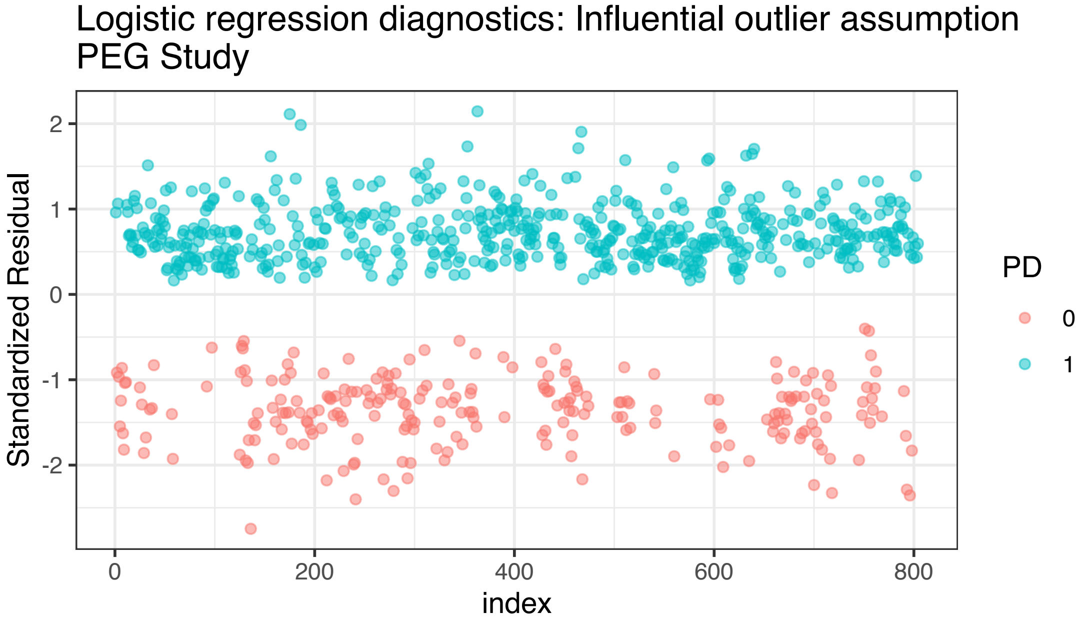
Logistic regression diagnostics: Influential outlier assumption
PEG Study
Standardized Residual
index
PD
0
1
2
1
0
-1
-2
0
200
400
600
800
