# Supplementary material for: DNA methylation biomarker for cumulative lead exposure is associated with Parkinson’s disease
Source: Clin Epigenetics. 2021 Mar 22;13:59. doi: 10.1186/s13148-021-01051-3 (PMC7983295; doi:10.1186/s13148-021-01051-3)
Supplement: Supplementary file 3 — Additional file 3: Table showing DNAm estimated cumulative lead exposure and PD risk in PEG, with and without controlling for smoking. [file 13148_2021_1051_MOESM3_ESM.docx]

**Supplemental Materials**

**Supplemental Figure 1.** Box plots of the DNAm lead-biomarkers by PD and stratified by study.

| **Supplemental Table 1. DNAm estimated cumulative lead exposure and PD risk, stratified by sex** | | | | | |
| --- | --- | --- | --- | --- | --- |
| **DNAm Lead** | **Strata** | **SGPD** | | **PEG** | |
|  |  | **OR (95% CI)** | **p-value** | **OR (95% CI)** | **p-value** |
| **Tibia** | Men Only | 2.48 (1.86, 3.34) | 1.1E-09 | 1.49 (1.02, 2.20) | 0.042 |
|  | Women Only | 1.67 (1.21, 2.33) | 2.3E-03 | 1.81 (1.17, 2.85) | 0.009 |
| **Patella** | Men Only | 0.58 (0.41, 0.82) | 2.5E-03 | 1.09 (0.69, 1.71) | 0.692 |
|  | Women Only | 0.60 (0.39, 0.91) | 1.7E-02 | 1.49 (0.75, 3.02) | 0.257 |
| Models adjust age and ancestry (PEG only) | | |  |  |  |

| **Supplemental Table 2. DNAm estimated cumulative lead exposure and PD risk in PEG, with and without controlling for smoking.** | | | |
| --- | --- | --- | --- |
|  |  | **OR (95% CI)** | **p-value** |
| **MODEL 1: adjusts age, sex, ancestry** | **DNAm Lead (Tibia)** | 1.60 (1.20, 2.15) | 0.001 |
| **MODEL 2: adjusts age, sex, ancestry, and smoking** |  | 1.58 (1.18, 2.12) | 0.002 |
| **MODEL 1: adjusts age, sex, ancestry** | **DNAm Lead (Patella)** | 1.19 (0.82, 1.73) | 0.356 |
| **MODEL 2: adjusts age, sex, ancestry, and smoking** |  | 1.18 (0.81, 1.73) | 0.401 |
